# Connexins: a myriad of functions extending beyond assembly of gap junction channels

**DOI:** 10.1186/1478-811X-7-4

**Published:** 2009-03-12

**Authors:** Hashem A Dbouk, Rana M Mroue, Marwan E El-Sabban, Rabih S Talhouk

**Affiliations:** 1 Department of Biology, Faculty of Arts and Sciences, American University of Beirut, Beirut, Lebanon; 2 Department of Human Morphology, Faculty of Medicine, American University of Beirut, Beirut, Lebanon; 3 Department of Molecular Pharmacology, Albert Einstein College of Medicine, Bronx, NY, USA; 4 Division of Life Sciences, Lawrence Berkeley National Laboratory, University of California, Berkeley, CA 94720, USA

## Abstract

Connexins constitute a large family of trans-membrane proteins that allow intercellular communication and the transfer of ions and small signaling molecules between cells. Recent studies have revealed complex translational and post-translational mechanisms that regulate connexin synthesis, maturation, membrane transport and degradation that in turn modulate gap junction intercellular communication. With the growing myriad of connexin interacting proteins, including cytoskeletal elements, junctional proteins, and enzymes, gap junctions are now perceived, not only as channels between neighboring cells, but as signaling complexes that regulate cell function and transformation. Connexins have also been shown to form functional hemichannels and have roles altogether independent of channel functions, where they exert their effects on proliferation and other aspects of life and death of the cell through mostly-undefined mechanisms. This review provides an updated overview of current knowledge of connexins and their interacting proteins, and it describes connexin modulation in disease and tumorigenesis.

## Introduction

A cell in its normal physiological context within a tissue perceives micro-environmental cues from soluble mediators, extracellular matrix (ECM), and neighboring cells. Adhesion molecules mediating cell-cell and cell-ECM interactions are multifunctional, providing structural support and links between the cell and its environment, and also organizing the cell's cytoskeleton as well as initiating and integrating signaling cascades [1,2].

Disruption of adhesion complexes, mainly adherens junctions, tight junctions, and gap junctions, leads to interference with normal tissue function, and may eventually lead to tumor development [3]. The adherens and tight junctions have been studied as independent entities, and together as key components of the apical junctional complex which regulates paracellular permeability (barrier function) and cell polarity, among other functions [4].

Among cell-cell adhesion molecules, intercellular communication by gap junctions is paramount for maintaining cellular homeostasis and function [5,6]. Connexins and connexin-like proteins, in vertebrates and invertebrates, pannexins and innexins, are proteins capable of forming gap junctions [7]. Twenty different connexin genes have been found in mice and 21 in humans [8], and these, *via *formation of gap junctions, allow direct exchange of small molecules between adjacent cells, including important signaling molecules such as Ca^2+^, IP_3_, and more recently siRNA [9,10]. Connexin structure is highly conserved; however certain cytoplasmic domains show variability between different isoforms and allow for different interactions and functions. The known repertoire of connexin associated proteins has recently expanded, and includes cytoskeletal elements, enzymes (kinases and phosphatases), adhesion molecules, and signaling molecules [11]. These connexin-associated proteins regulate and mediate both channel-dependent and -independent functions of connexins. In addition, connexin mutations, altered expression, and impaired gap junction function are characteristic of some diseases and neoplasms [12,13].

In this review, we present an updated overview of connexins, as well as the rapidly expanding list of connexin-associated proteins. We also discuss the various connexin functions, whether these are channel dependent or independent, and we attempt to document the inter-relationship between the connexin-associated proteins and these functions. In addition, we reveal the impact of disruption of gap junction and connexin function on the process of disease and cancer development.

## The connexin family

Gap junctions are clusters of channels that join two cells together and consist of building blocks of two connexons or hemichannels, one contributed by each of the communicating cells. Each connexon or hemichannel is formed of a complex of six connexin proteins. Such junctions allow intercellular exchange of molecules less than 1.5 kDa in molecular weight [14,15]. Most cells of normal tissue, except skeletal muscle cells, erythrocytes, and circulating lymphocytes, communicate via gap junctions. This is an indication of the pivotal roles that proper connexin gene expression, protein levels, and protein function play in regulating various physiological phenomena including, growth, differentiation, developmental signaling, and cell death [5,16-18].

### Connexin gene

Most connexins, with very few exceptions [19], share a common gene structure, consisting of two exons separated by one intron sequence. The intron, which is of variable length, is situated within the 5'-untranslated region, while the second exon contains the connexin coding sequence, and the 3'-untranslated region [20]. In noted exceptions, the coding sequence is interrupted by introns, and this is observed in a few mouse and human connexins. In addition, connexin genes may have different tissue-specific promoter usage patterns resulting in different transcription start sites and a different 5'-UTR [8].

The sequencing of connexin genes began in the 1980s, and most of the remaining, rodent and human connexin genes, were sequenced in recent years. Evolution studies on connexin sequences have shown that they are chordate-specific and highly conserved [21,22]. Invertebrates do not express connexins and display direct cell-cell communication via a family of proteins termed innexins, which play a connexin-like role, even though they lack sequence homology to connexins [23,24]. Pannexin genes, innexins homologues, have since been discovered in the genomes of higher vertebrates, including humans, where they are expressed in a variety of tissues, with seemingly significant functions, most notable in the brain and in mediating immune response to pro-inflammatory stimuli through ATP-dependent activation of the inflammasome complex [7,25].

The various connexin isoforms are differentially-expressed, displaying spatial and temporal specificity modulated by a variety of transcription factors, including the Sp transcription factors (Sp1 and Sp3), Activator protein 1 (AP-1), as well as members of the Jak/STAT pathway [26,27]. In addition, other cell type specific transcription factors regulate connexin gene expression and these include Nkx2, HNF-1, Mist1, NF_K_B and others [27,28]. In the past few years it has also become apparent that epigenetic processes are involved in regulating connexin gene expression. In addition to histone modifications and DNA methylation, recently, microRNA species have been reported as key regulators of Cx expression [29,30].

### Connexin protein

The connexin protein is composed of nine main domains, of which the N-terminus, the two extracellular loops (stabilized by intramolecular disulfide bridges), and the four transmembrane domains are highly conserved among different isoforms. In contrast, the cytoplasmic loop domain and the C-terminus domain are divergent and variable in length and sequence, allowing for the functional differences among the different connexins and the connexon types [31-33]. The N-terminus, cytoplasmic loop, and C-terminus are located in the cytosol, and this allows for the interaction of these domains, the C-terminal domain in particular, with connexin-interacting proteins such as catenins and others, which are essential for modulating connexin half-life, activity and functions [11]. The connexin nomenclature is according to one of two systems: (i) The first is based on a "number" system whereby the molecular weight predicted from the cDNA sequence of the connexins denotes the type of the connexin. For example, Cx26, Cx32, Cx43 refers to the connexins with molecular weight 26 kDa, 32 kDa and 43 kDa respectively [34], and (ii) the second is based on sequence similarity and length of the cytoplasmic domain of the connexins, thereby classifying them into α, β, and γ subgroups [35]. In this latter the connexins are designated as "GJ" for gap junction and serially numbered in the order of their discovery within each subgroup. For example, Cx43 was the first connexin discovered in the α subgroup and was designated as Gja1, while Cx32 was the first in the β subgroup and was designated as Gjb1. However, until recently there were discrepancies between both nomenclatures. Further alignment between the two nomenclature systems, to alleviate the discrepancies, was recommended [36], and later adopted.

#### Synthesis and maturation

Connexins are typically translated into polypeptide sequences by ribosomes attached to the endoplasmic reticulum (ER), similar to other transmembrane proteins, followed by sequential release of the protein (co-translationally) into the ER lumen through the Sec61 or translocon, until completion of translation [37,38]. This is followed by the folding of the connexin protein while still in the ER.

Connexons are formed of six connexin proteins, which assemble into a hemichannel with either identical connexin subunits (homomeric) or different connexins (heteromeric). In addition, gap junctions may be homotypic, when two identical connexons assemble, or heterotypic, when two dissimilar connexons assemble between the two interacting cells. Since many cells express multiple connexin isoforms, the possibility of heteromeric connexon hemichannels is highly significant in raising the number of potential combinations of the approximately twenty available connexin types. Also, channels formed of heteromeric connexons have different properties from those of homomeric channels of the constituent connexins, and the properties of such heteromeric channels can be manipulated by regulating the ratio of constituent connexins, thereby allowing for critical regulation of the permeability and conductance of gap junctions [39]. It has been suggested that C-terminal peptide sequences in connexins may act as assembly signals regulating principal connexin subunit recognition; moreover a selectivity signal regulating specific assembly of heterotypic connexons is located in the amino-terminus (at the first transmembrane domain or the first extracellular loop) of the connexin polypeptide sequence [38]. Thus, the control of connexin oligomerization occurs at two levels, the structural compatibilities of the connexin types, and the cellular mechanisms that may interfere in the interaction between compatible connexins [40].

**Figure 1:**
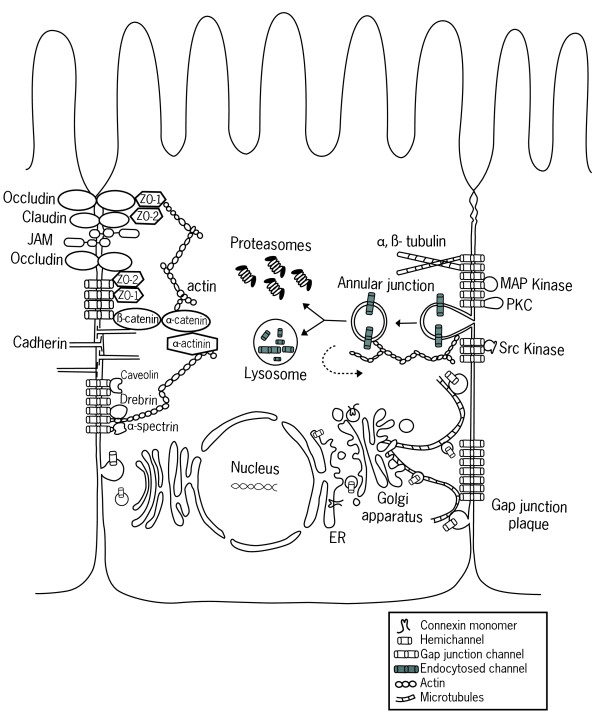
**Life cycle and protein associations of connexins**. Connexins are synthesized on ER-bound ribosomes and inserted into the ER cotranslationally. This is followed by oligomerization between the ER and trans-Golgi network (depending on the connexin type) into connexons, which are then delivered to the membrane via the actin or microtubule networks. Connexons may also be delivered to the plasma membrane by direct transfer from the rough ER. Upon insertion into the membrane, connexons may remain as hemichannels or they dock with compatible connexons on adjacent cells to form gap junctions. Newly delivered connexons are added to the periphery of pre-formed gap junctions, while the central "older" gap junction fragment are degraded by internalization of a double-membrane structure called an annular junction into one of the two cells, where subsequent lysosomal or proteasomal degradation occurs, or in some cases the connexons are recycled to the membrane (indicated by dashed arrow). During their life cycle, connexins associate with different proteins, including (1) cytoskeletal components as microtubules, actin, and actin-binding proteins α-spectrin and drebrin, (2) junctional molecules including adherens junction components such as cadherins, α-catenin, and β-catenin, as well as tight junction components such as ZO-1 and ZO-2, (3) enzymes such as kinases and phosphatases which regulate the assembly, function, and degradation, and (4) other proteins such as caveolin.

Connexin oligomerization into connexons was initially reported to occur in the ER, however, some exceptions have been shown in which connexins remain in the monomeric form (i.e. single connexin proteins) upon reaching the Golgi apparatus where they oligomerize into connexons [41,42]. Therefore, it has been suggested that connexin oligomerization occurs sequentially throughout the transport of the connexins within the ER till the trans-Golgi network, where oligomerization is completed (Figure 1) [13,38]. An exception to this pathway is Cx26 which may be either co- or post-translationally transported into the ER [43] or directly to the plasma membrane [44], without passing through the Golgi apparatus [45].

#### Transport to the cell membrane

Upon completion of connexin oligomerization, connexon hemichannels are packaged into vesicles (between the ER and the trans-Golgi network) and delivered to the membrane. This connexon transport may either be microtubule dependent or independent, and these include actin-mediated transport as well as other unidentified mechanisms [38,46-48]. Even though connexon insertion in the plasma membrane has been suggested to be random, microtubules may mediate targeting of these gap junction hemichannels to specific membrane domains or regions, especially those containing adherens junction components [49].

Following connexon insertion into the plasma membrane, the hemichannel may remain uncoupled, where it has been shown to play possible roles in the life of the cell [50]. However, the common pathway is for inserted and apposing connexons to interact *via *their extracellular loops to form intercellular channels. The individual channels aggregate in the membrane to form plaques, and these are termed gap junctions (Figure 1) [51].

#### Post-translational modifications

Connexins may undergo various types of post-translational modifications, including phosphorylation, hydroxylation, acetylation, disulfide binding, nitrosylation, and palmitoylation [52-55]. The most studied of these connexin post-translational modifications is phosphorylation of connexins on various residues. These phosphorylation events are essential in the proper control of formation and modulation of function of gap junction channels, and they have been observed in at least nine connexins (Cx31, Cx32, Cx37, Cx40, Cx43, Cx45, Cx46, Cx50, and Cx56), while others, such as Cx26 remain unphosphorylated. Connexin phosphorylation by different kinases such as Src, PKC, and MAPKs is required for and affects connexin/connexon trafficking, assembly and disassembly, degradation, and gating (rapid opening and closing) of gap junction channels [56,57]. The effect of phosphorylation on channel gating is very specific, as the phosphorylation of a connexin isoform such as Cx43 on different residues by the same kinase may lead to opposite effects with respect to enhancing or inhibiting gap junction function or gap junction intercellular communication (GJIC) [18]. The phosphorylation events also affect other cellular functions of connexins that are independent of gap junctions, including the control of growth and proliferation (These are elaborated upon in section III of this review).

In addition, the association of connexins with other interacting proteins also plays an important role in the stability and function of gap junctions [11,58].

#### Half life and degradation

Since connexin proteins have a very short half-life not exceeding few hours, the synthesis and delivery to the membrane of new connexin proteins is coupled to simultaneous gap junction internalization and connexin degradation [38,59]. It has been suggested that newly delivered connexons localize to the periphery of existing gap junctional plaques, whereas the "old" connexons that are to be degraded are usually present at the center of the gap junctional plaque [60].

The mechanism of gap junctional internalization is through the formation of annular junctions, which are large double-membrane vesicular structures that could contain the entire gap junction, or a fragment of it, and transport it, from cell-cell boundaries, into one of the two interacting cells [13,61]. Following gap junction internalization, these complexes undergo preliminary degradation in the annular junctions, leading to disassembly of gap junctions and connexons into individual connexins [62]. The connexin proteins undergo complete degradation through either proteasomes or lysosomes, with each of the two degradation pathways having different roles. As such, the proteasome degradation pathway may regulate connexin stability and internalization from the cell membrane, but this effect is suggested to be indirect, possibly through regulating the turnover of Cx-associated proteins, such as ZO-1 [63,64]. On the other hand, lysosomal degradation plays a more central role in connexin protein degradation, either before transport to the membrane or after incorporation in the membrane but before the formation of functional gap junctions [13,38,64,65].

## Connexin associations

Connexon docking and channel assembly at the cell surface is the preliminary step towards proper cellular communication and formation of gap junctions. However, connexin proteins do not act in isolation in gap junctional complexes, but rather interact with many associated proteins that play essential roles in regulating the assembly, function, and life-span of connexins [11,58].

The number and diversity in connexin associating partners has increased steadily over the past years, implicating tight and adherens junctional proteins in the complex, as well as protein phosphatases and kinases, in addition to cytoskeletal elements, in the regulation and control of the gap junction complex [11,18,51,58,66,67]. The main interacting partners of connexin proteins are cytoskeletal elements, junctional proteins, and enzymes (Figure 1).

As connexin 43 is the most studied connexin protein, due in part to its expression in a wide variety of different tissues, it was of inherent interest to study its interactions with cytosolic proteins. The C-terminal region of Cx43 can interact with many proteins, including kinases, phosphatases, membrane receptors, cell signaling and scaffolding proteins. The N-terminal, cytoplasmic loop, and some membrane-spanning domains of connexins also interact with a variety of proteins, some of which bind the C-terminus as well [11,66].

### Connexin interactions with cytoskeletal elements

Interactions of connexins with cytoskeletal elements are essential in transporting connexins to the membrane and providing a mean for their rapid turnover and replenishment.

#### Microtubules

Co-immunoprecipitation experiments revealed that Cx43 sediments with microtubules and bioinformatics analysis confirmed this result by unraveling a tubulin-binding domain (^234^KGVKDRVKGL^243^) in Cx43 [68]. It has been confirmed that Cx43 directly binds to α- and β-tubulin and that microtubules at the cell-periphery co-localize with Cx43-based gap junctions. The 35 juxtamembrane amino acids of the C-terminal tail were found to be necessary and sufficient for this interaction [68,69]. Furthermore, multiple alignment studies from our laboratory [18] have revealed a tubulin-binding site in connexin 46 and in xenopus oocyte connexin 41[70].

This interaction of connexin proteins with microtubules has been shown to be essential in allowing directed transport of newly synthesized connexin hemichannels to the plasma membrane. That microtubules also interact with other junctional molecules such as cadherins allows increased specificity in the targeting of connexins to the adherens junction, and this directs the delivery of newly-synthesized connexons to areas of pre-existing adherens junctions [49].

#### Actin, α-spectrin, and drebrin

Interactions of connexins with the actin cytoskeleton and associated proteins serve to stabilize gap junctions at the plasma membrane. Immuno-histochemical analysis has shown that Cx43 and actin colocalize in a variety of cell types [71]. In addition, studies have shown that the membrane cytoskeletal protein, α-spectrin co-precipitates with gap junctional complexes, possibly *via *interaction with ZO-1 [72]. The existence of various isoforms of α-spectrin requires functional studies of each with respect to their effect on gap junction function. For example, an alternatively spliced αII-spectrin isoform has been shown to play a role in transducing stress-induced signaling by forming a physical link between JNK stress-induced signaling pathway, and GJ function, to influence intercellular communication under stressful conditions [73].

A novel connexin-interacting cytoskeletal protein is drebrin ("developmentally regulated brain protein"), an actin-binding protein involved in mediating cellular polarity and formation of stabilized plasma membrane domains [74], which has been shown to interact with the carboxy-terminal end of Cx43. This interaction allows the stabilization of Cx43 gap junctions at the membrane, and as such may control its life cycle, as siRNA depletion of drebrin led to the inhibition of cell coupling, and to internalization and degradation of Cx43 [75].

### Connexin interactions with junctional proteins

The interaction between gap junctional proteins and proteins of the adherens and tight junctions is a clear indication of the interplay between these cell adhesion complexes. The inter-connectedness between the three junctional complexes, both direct interactions and indirect interactions through their associated proteins, has given rise to the concept of formation of large protein intercellular complexes containing multiple junctional molecules. The interaction between connexins and other adhesion proteins might be involved in regulating connexin assembly, trafficking, turnover and channel gating, in that these complexes may form a regulatory backbone for various stages of the connexin life cycle, including membrane insertion, localization, and gap junctional plaque formation [11,58].

#### Interactions with tight junction proteins

Studies have shown that several growth factors that regulate GJIC also regulate tight junction permeability, while others have shown that connexins and tight junction tetraspan membrane core proteins, claudins and occludins [76,77] co-immunoprecipitate, and that gap junctions may even regulate the expression and function of tight junction proteins [11,58,78-80].

##### ZO-1 and ZO-2

The tight junction membrane-associated guanylate kinase protein, zonula occludens-1 (ZO-1) is involved in the organization and trafficking of gap junctions. In a study by Laing *et al*. [81], ZO-1 was shown to regulate Cx43-mediated gap junctional communication in osteoblastic cells by altering the membrane localization of Cx43. The study suggested that ZO-1 mediates the delivery of Cx43 from a lipid raft domain to gap junctional plaques, which is an important regulatory step in gap junction formation. The C-terminal residues of Cx43 and Cx45 interact with the second PDZ domain of ZO-1, and this binding can recruit regulatory proteins into the gap junctions [63,69,82-84]. Another potential role for the interaction of ZO-1 with Cx43 is to act as a scaffold for other proteins, thereby bringing them into close contact with either the gap junction proteins themselves or with molecules that pass through the gap junction plaques. For example, ZO-1 can bind to catenins in epithelial cells and act as an adapter for the transport of connexin 43 during gap junction formation [85]. Barker *et al*. [86] revealed that association between ZO-1 and Cx43 is increased during remodeling of cardiac gap junctions, possibly implicating ZO-1 in gap junction turnover during cardiac development and possibly during disease progress. In addition to Cx43, other connexins encode putative PDZ binding sequences at their C-termini, raising the possibility that other Cx's might be associating with ZO-1 [11,87].

Moreover, another connexin, Cx32, colocalizes with tight junction proteins ZO-1 and ZO-2 in rat hepatocytes, and small gap junction plaques were found within tight junction strands. It was suggested that Cx32 can participate in the formation of functional tight junctions and in actin organization [78]. Recently our laboratory has shown that Cx30, Cx32, and Cx43 associate with ZO-2, as well as other Cx-associated proteins, during mammary epithelial cell (MEC) differentiation, and that these associations are crucial for MEC differentiation [88].

Furthermore, the associations between Cx43 and ZO-1 and ZO-2 proteins were shown to be under cell-cycle stage-specific control. Connexin 43 interacts preferentially with ZO-1 during G_0 _stage, whereas the interaction with ZO-2 is approximately the same during the various stages [89]. This may signify a greater level of complexity in the connexin-zonula occludens interactions and their impact on cellular functions.

##### Occludin and claudin

Occludin, like the connexins, is also a four transmembrane domain protein. Recent studies using cultured hepatocytes showed that occludin is also associated, and co-immunoprecipitates, with gap junctional proteins, in particular connexin 32 [77]. Examination of freeze-fracture replicas of these cells shows many small gap junctional plaques localized near tight junctional strands, and co-immunoprecipitation studies on cultured hepatocytes western blotted with Cx32 provided evidence for an association between occludin and Cx32 [51]. Cx26 has also been reported to interact with occludin in polarized sheets of the human intestinal cell line T84 [90]. An additional tetraspan family of proteins, the claudins, has also been found to associate with connexins. Kojima *et al*. [77] found that along with an association with occludin, Cx32 also associated with claudin-1 in hepatocytes, while Cx26 [91] has been shown to colocalize with claudin-14 in human airway epithelial cell lines and to have a gap junction-independent role in maintaining the proper functioning of tight junctions.

#### Interactions with adherens junction proteins

Since it was established that important cellular processes such as cell proliferation and growth are partly regulated by connexin-cadherin interactions, and with the notion that connexins modulate adherens junction formation, and adherens junctions participate in gap junctional assembly [92], it has become pivotal to study connexin-adherens junction associations. This intimate linkage between gap junction and adherens junction formation reflects the close membrane-membrane apposition required for junction formation. It also provides evidence for the assembly of multi-adhesion molecule complexes between cells and how that mediates proper intercellular interactions in tissues to allow enhanced and optimal signaling between cells.

##### Cadherins

A number of reports addressed connexin co-localization with cadherin proteins. Wei *et al*. [93] reported the co-localization and co-immunoprecipitation of Cx43 protein with N-cadherin, p120 and other N-cadherin associated proteins at regions of cell-cell contact. Matsuda *et al*. [94] also showed an association between Cx43 and N-cadherin, and this interaction required Rac1 and RhoA-mediated signaling downstream of N-cadherin membranous localization, leading to targeted connexin delivery and insertion at the membrane. Moreover, the upregulation of E-cadherin-dependent cell-cell contacts increased GJIC in mouse epidermal cells [95], while anti-N-cadherin antibodies prevented both adherens junctions formation and GJIC [92]. A study by Xu *et al*. [96] revealed that association between cadherins and connexins is not only important for structural purposes, but that gap junction mediated dye coupling is inhibited in a knock-out N-cadherin mouse model.

Recently, Huang *et al*. [97] demonstrated linkage between adherens and gap junctions by treatment of rat adenocortical cells with 18*β*-glycyrrhetinic acid, a gap junction inhibitor. This treatment reduced the immunoreactivity of these proteins in a time- and dose-dependent manner, and caused the gap junction and adherens junction to separate longitudinally from the cell-cell contact sites, indicating the structural interdependency of these two junctions.

##### Catenins

Cx43 interacts with *β*-catenin, a versatile protein with adhesive and transcriptional functions, and the connexin-*β*-catenin complex colocalizes mainly at the cell membrane. This finding [98] raised the question as to whether Cx43 is regulating *β*-catenin signaling by sequestering it at the membrane. The ability of Cx43 to modulate *β*-catenin signaling suggests that connexins, in addition to their classical channel forming role, might be involved in regulating cell growth by yet another mechanism. For example, p120 catenin-related protein was co-localized with Cx43 and N-cadherin in mouse neural crest cells [96]. In addition, both E-cadherin and α-catenin were co-localized with Cx26 and Cx32 during gap junction reappearance in mouse hepatocytes [99]. Connexins 30, 32, and 43 have been shown to associate with both α-catenin and *β*-catenin in mammary epithelial cells, and this interaction was proposed to sequester *β*-catenin away from the nucleus in differentiation-permissive conditions [88]. In addition to the documented interaction of *β*-catenin with connexins, and its implications for regulating function, studies suggest a role for α-catenin/Cx43 interaction in the assembly and trafficking of the gap junction proteins to the membrane [100].

Given that N-cadherin and catenins are co-assembled in the endoplasmic reticulum and Golgi compartments, and in the light of reported interaction of Cx43 with the former proteins, the possibility of Cx43 assembly as part of a multi-protein complex that regulates gap junction and adherens junction formation is raised and future studies are needed to address this question.

### Connexin interactions with enzymes

The post-translational modification of connexin proteins, mostly represented by phosphorylation processes, implies that connexins interact significantly with various protein kinases, as well as phosphatases. This phosphorylation is essential for a variety of the connexin family members during their assembly into gap junctions [101]. It has also been noted that gap junctional communication may be suppressed by the action of a number of protein kinases, growth factors and oncogenes, and that this suppression correlates often with mitogenesis [102].

#### Tyrosine kinases

Studies have shown that Cx43 is a v-Src substrate [103]. Mutation of the putative v-Src phosphorylation site results in lack of gap junction closure by v-Src in Xenopus oocytes [104]. Further studies showed that phosphorylated Tyr265 in Cx43 forms a docking site for the SH2 domain of v-Src and that the SH3 domain of v-Src can bind to a proline rich stretch in Cx43 [105]. In addition, Tyr247 can be phosphorylated by v-Src. Accordingly, a model was proposed in which (i) v-Src binds to Cx43 *via *a SH3 domain/proline-rich motif association; (ii) Src then phosphorylates Cx43, mainly on Tyr265; and (iii) subsequent docking of the SH2 domain of Src to Cx43 Tyr265 increases affinity and positions Src for (iv) Tyr247 phosphorylation leading to channel closure [106]. Other reports by Giepmans *et al*. [107] and Toyofuku *et al*. [82] also revealed that Tyr265 phosphorylation by c-Src is most likely involved in interaction of Cx43 with ZO-1. Moreover, phosphorylated Cx43 binds to c-Src *via *the SH2 domain, competing with ZO-1 binding to the connexin protein [82,106].

#### Serine/threonine kinases

The involvement of serine/threonine kinases in gap junction regulation has also been well documented. For example, PKC acts on Ser368 of Cx43, resulting in inhibition of GJIC [57]. Interestingly, PKCε, which has been implicated in inhibition of GJIC downstream of the fibroblast growth factor-2, has been reported to co-immunoprecipitate and co-localize with Cx43 in cardiomyocytes [108]. Similar results have been described for the PKCγ isotype acting downstream of the insulin-like growth factor receptor-I [109]. However, an upregulation of GJIC occurs by PKCα concomitant with Cx43 phosphorylation [110], and as such, maximal Cx43 phosphorylation and GJIC are observed during mammary gland differentiation and lactation [111,112]. Another study by Seo *et al*. [113] showed that Cx 26, 32, and 43 are regulated by MAP kinases during differentiation of rat mammary epithelial cells. Interestingly, MAPK was shown to interact with Cx43 and mediate its phosphorylation, however, the effect of this phosphorylation on gap junction gating and function remains controversial [80]. The seemingly contradictory effects of PKCs on Cx43 and GJIC likely reflect the complexity of channel regulation, where it may regulate and alter the permeability of gap junctions and hemichannels [114].

#### Phosphatases

Other reported partners for connexins include both serine/threonine and tyrosine phosphatases but the functions of these, although apparently of significance in the regulation of connexin functions, are yet to be clearly defined and determined [115]. Most studies have been performed with Cx43, showing a direct interaction with several defined and undefined protein phosphatases [67,116,117], although other connexins are also affected by various phosphatases. The role of serine/threonine phosphatases is to limit gap junctional conductance, and these have been shown using various phosphatase activators and inhibitors [118]. These effects of phosphatases have been observed in cardiac myocytes upon conditions of hypoxia or metabolic starvation [118], and also upon stimulation of T51B rat liver epithelial cells with EGF [119]. Similarly, phosphatases have been shown to function upon treatments of cells with known gap junction inhibitors such as 18β-glycyrrhetinic acid [120] and 2,3-Butanedione monoxime [121]. On the other hand, tyrosine phosphatases may have a role in enhancing GJIC, as inhibition of the activity of these enzymes significantly decreased GJIC in various cell types [122,123].

### Other connexin-interacting partners

Among newly-discovered interacting connexin partners, plasma membrane ion channels, membrane transport proteins and receptors have been shown to interact directly or indirectly with connexins, and these include aquaporin-0 and acetylcholine receptors, among others [11,124]. Various connexins have also been shown to interact with calmodulin, which has been implicated in control of connexin channel gating [11,125]. Furthermore, the calcium/calmodulin-dependant kinase II (CaMKII) interacts with and phosphorylates Cx36 and mediates channel gating in inferior olive neuronal cells [126]. CaMKII has also been shown to mediate enhanced gap junctional coupling, mostly through Cx43, in response to high extracellular K^+ ^in mouse spinal cord astrocytes [127]. Thus this interaction of connexins with CaMKII may have a general regulatory role in neuronal signal transmission, with a role in electrical coupling in addition to the defined role of CaMKII in chemical synaptic transmission [128,129]. Other interactions include cholesterol, which was shown to interact with gap junction channels in different ratios during fiber cell differentiation in embryonic chicken lens [130].

Cx43 co-localized with Cox-2 in intestinal adenomas, providing a possible role for transmission of signals between stromal and epithelial cells of the tumor, as their expression localized to myoepithelial cells rather than tumor epithelial cells. Worth noting is that both Cx43 and Cox-2 are nuclear *β*-catenin target genes. Together, the implication of *β*-catenin regulation and its association with connexins in stromal cells and how this regulates tumor growth, as adenomas, raise significant questions that remain unanswered [131]. Recent reports describing localization of Cx43 to mitochondria has shown that heat shock protein 90 (Hsp90) and translocase of the outer mitochondrial membrane (TOM) are Cx43-interacting partners, which help in transport and insertion of Cx43 in the inner mitochondrial membrane [132,133]. Other interactions include that with caveolin-1 which binds to Cx43, and thus provides a potential framework for the regulation of connexin internalization and gap junction degradation [134].

## Connexin functions

The increasing interest in connexins has revealed a variety of novel functions that have been attributed to these proteins. Also of interest is the ever-increasing number of discovered Cx-associated proteins which sheds light on the complexity of the interactions of these proteins, allowing connexins to play major roles in many cellular and developmental functions [11]. In addition, novel discoveries concerning the activity of connexin hemichannels, as well as connexins by themselves -independent from both hemichannels and gap junctions- have increased the scope of connexin roles in the cell [5,50,135].

Connexins have been shown to play essential roles in normal development and differentiation of a variety of tissues. Connexins and their associated proteins are implicated in mammary gland differentiation, with their levels and phosphorylation status being modified throughout the differentiation process, as well as through modulation of their associated proteins [88,112]. Furthermore, connexins are central for the normal functioning of body organs, such as the synchronized contraction of the heart muscle [136-138], as well as in essential physiological processes such as tissue inflammation and repair [139]. Also, the loss of connexins, or the existence of mutations affecting their normal functions, has been implicated in a variety of diseases and disorders, including cancers [12,13].

### Gap junction dependent functions

Gap junction intercellular communication (GJIC) represents a central conduit of ions, essential metabolites, and second messengers, such as Ca^2+^, cAMP, cGMP, and IP_3_, between adjacent cells. In past decades, GJ contribution to the organism homeostasis was construed as passive. However, studies have shown that gap junctions undergo complex regulation, and play an active role in intercellular signaling and communication.

#### Regulation of gap junction function

Gap junction channels, depending on cellular needs and conditions, alternate between "closed" and "open" conformations. These changes are dependent on and are regulated by various mechanisms including calcium concentration, pH, transjunctional potential, and protein phosphorylation. It has been shown that calcium-dependent cellular processes and events might be regulating gap junctional function, and thus increasing the significance of connexin interactions with calcium-affected molecules such as calmodulin [125,126,140]. Moreover, intracellular pH can also modulate the gating of gap junction channels. In fact, regulatory sites that respond to pH levels within gap junctions were found in the intracellular loop and carboxy terminus domains of the connexin proteins, a region which shows little sequence homology between different connexins [141,142]. Hence, differential composition of the gap junction channels is an important determinant of the response to pH in different cell types [143]. Gap junction conductance (i.e. opening and closure) and selective permeability to ions and metabolites were also shown to respond to trans-membrane voltages whereby large trans-junctional voltages were shown to close the channels, possibly through the C-terminus interacting with the pore of the channel [144,145]. This voltage-mediated regulation of GJIC controls the rate of passage of ions and molecules (such as cAMP) between cells while maintaining electrical coupling [146]. In addition, changes in connexin phosphorylation also affect GJIC. This effect can occur through a change in the number of gap junctions at the cell-cell interface by controlling connexin trafficking and degradation, by changing the efficiency of passage of certain molecules through GJs, or by completely closing or opening the channel for passage of molecules [147-149].

#### Significance of gap junctional communication

An array of studies illustrated the important contribution of GJIC to developmental and regulatory events such as embryonic growth, bone modeling, alveolar differentiation, central nervous system signaling and neural function in the developing central nervous system [150-155]. In addition, studies pointed to the significant role of GJIC in maintenance of tissue homeostasis through processes such as synchronized contraction of cardiac muscle cells [137].

Tissue differentiation is an essential and continuous process, occurring during both embryonic and adult life of the organism. Various studies have shown that proper GJIC is essential for the differentiation of various tissues, including the mammary gland, lens, bone marrow, sertoli cells, adipocytes, and other cells and organs [111,156-160]. Previous studies conducted in our laboratory using the CID-9 heterogeneous mouse mammary cell strain established that enhanced GJIC induced partial differentiation in mammary epithelial cells in the absence of an exogenously provided basement membrane [111]. Interestingly, restoration of gap junctional communication in colon cancer cells leads to re-establishment of a differentiation phenotype, and the observed re-differentiation of these cells correlates with increased levels of connexin43 protein and its phosphorylation status [161].

Gap junction mediated communication can exist between different cell types. Many reports have characterized heterocellular gap junctions in vivo and in vitro, and attributed important regulatory roles for heterocellular GJIC. For example, a study by Abraham *et al*. [150], revealed an important role of heterocellular gap junctional communication between Type I and Type II epithelial alveolar cells to promote epithelial differentiation in the rat lung. Previous studies have also reported the importance of heterocellular GJIC between cardiac myocytes and surrounding fibroblasts [162,163], between germ cells and Sertoli cells in the testis [164], and between neuronal and glial cells in the nervous system [165]. Moreover, Veitch *et al*. [166] recently reported that heterocellular GJ exist in the growing murine uterus between oocytes and granulosa which are functionally coupled by homotypic gap junctions composed of Cx37. In addition, our laboratory has recently shown that gap junction mediated heterocellular interaction between myoepithelial-like cells and mammary epithelial cells can induce differentiation of the latter and leads to concomitant assembly of connexins into gap junction proteins, and in association with α- and *β*-catenins and ZO-2 proteins [88].

Gap junctions are also implicated in cell death. This is due to the "bystander effect" in which gap junctions spread a death signal between dying cells and those adjoining them and this may be mediated by Ca^2+ ^influx between the cells. Such a role of gap junctions in the spread of injury is observed in the brain following hypoxia-ischemia [167]. However, gap junctions have also been shown to "rescue" dying cells by the passage of substrates such as ATP, glucose, and ascorbic acid, as well as by inhibiting the passage of cytotoxic agents such as nitric oxide and others [5,167,168].

### Hemichannel dependent functions

Connexin hemichannels have overcome their classification as simple structural precursors of gap junctions. When discovered, hemichannels were thought to be a by-product of connexin over-expression in certain cell types, such as oocytes, however successive studies have located these structures in multiple cell types and in cultured cells, and even in isolated embryonic stem cells [155,169,170]. Hemichannels are now the subject of many studies that illustrate their role in various physiological conditions [50].

#### Regulation of hemichannel function

Hemichannels are usually closed upon reaching the cell membrane however their functional state is regulated by various physiological conditions, due to both intracellular and extracellular factors [171]. The regulation of hemichannels is through extracellular changes in ionic concentration [172,173], membrane depolarization [174], metabolic inhibition [55,175], and mechanical stimuli [176].

#### Roles of hemichannels in cells

Hemichannels function in various aspects of cell life, including calcium signaling, cell proliferation and death, as well as the normal functioning and development of various cell types [50]. For example, neurite outgrowth in PC12 cells upon stimulation with nerve growth factor was mediated by hemichannels through the release of ATP which interacts with purinergic receptors to induce growth, and possibly affect neuronal differentiation [177]. In addition, hemichannels are involved in the movement of NAD^+ ^into and out of cells, reversibly, which may regulate Ca^2+ ^concentrations through the CD38 transmembrane glycoprotein [178]. Hemichannels have also been shown to exist in heart ventricular myocytes, where they have an osmoregulatory role, with both negative and positive potential impacts with respect to mycordial infarcts and cardiac physiology [175,179].

Furthermore, hemichannels, similar to gap junctions, play a role in cell survival and cell death. The effect of hemichannels in mediating cell death is, similar to GJIC, observed during ischemic injury in different tissues leading to significant ionic deregulation in cells [167,180]. Hur *et al*. [181] showed that Cx43 hemichannels enhanced and accelerated cell death following staurosporin treatment. Also, Cx43 hemichannels have been shown to play a role in the transduction of survival signals, where treatment of osteocytes and osteoblasts with biphosphonates leads to hemichannel-mediated activation of Src kinase and ERK. This effect, mediated both by the hemichannel pore activity and the C-terminal domain, leads to ERK activation and attenuation of osteoblast cell death [182]. The recent discovery of pannexins, their hemichannel forming ability, and their co-existence with connexins in vertebrate cells suggest that data previously ascribed to connexin hemichannels should be re-considered [7].

### Gap junction- and hemichannel-independent functions of connexins

In addition to their multiple roles *via *exchange of molecules, whether through gap junctions or hemichannels, connexins by themselves also play various roles independent of their channel-forming properties. Such connexin functions are mediated through their multiple interacting partners, independent of channel formation, and subsequently lead to the modulation of gene expression resulting in a wide range of effects [135]. The first of such reports was by Lee *et al*., [183] which identified Cx26 as a putative tumor suppressor. This was later confirmed, showing that Cx26 inhibits cell migration and invasion in a gap-junction independent mechanism in the MDA-MB-435 tumor cell line through regulation of β1-integrin and MMP levels [184], as well as reverses the malignant phenotype of MCF-7 breast cancer cells [185]. Furthermore, the tumor suppressive properties of connexins has been shown for various connexins, including Cx32, which suppressed growth, invasion, and metastasis of renal cell carcinoma cell lines, and this was through various modulators including Src, tight junction proteins, VEGF and others [186]. In addition, controlled expression of Cx43 prevented cell growth independent of its channel-forming properties, but via the association of its C-terminal domain with proteins such as ZO-1 and c-Src [187]. In this respect, the ability of connexins to regulate gene expression independent of their channel functions has been recently documented [188]. Multiple studies have shown that alteration of connexin expression, whether through over-expression or deletion, leads to changes in gene expression in multiple pathways and cellular functions, including transcription, metabolism, cell/cell and cell/ECM adhesion, cellular signaling, transport, and cell cycle and division [189]. One such mechanism is through connexin-responsive elements (CxRE), where connexins and gap junctional communication induce differential recruitment of sp1 and sp3 transcription factors to the CxRE through the ERK/PI3K pathway, and this regulates expression of genes having this promoter element [190,191]. This effect of connexins on gene expression has also been observed in many studies re-expressing connexins in connexin-deficient tumors, where these affect the characteristics (growth and tumorigenicity) of the tumor cells via gap junction-dependent and independent mechanisms. This includes regulation of gene expression of proteins involved in the various processes, such as MMPs and TIMPs [184], S-phase kinase-associated protein 2 [192], and p21 [193].

Cell differentiation can also be mediated by gap junction-independent mechanisms of connexins. As such, it has been shown that Cx45.6 stimulates lens cell differentiation regardless of its channel-forming capability, as the C-terminal domain of the same connexin is sufficient to induce differentiation [194]. A study by Xu *et al*. [195] has shown that Cx43 mediated directional motility of cardiac neural crest cells independently from channel-forming functions and through its association with actin-binding proteins such as vinculin and drebrin. Similarly, embryonic neurons were shown to migrate using Cx43 and Cx26 gap junctions to provide cytoskeletal contact points with radial fibers, and this effect did not involve the exchange of molecules (i.e. functional gap junctions) between the two cell types at the point of contact [196].

As mentioned earlier, mitochondrial localization of Cx43 [132] is suggested to play vital, though still incompletely defined roles in various processes [133,197]. For example, mitochondrial Cx43 is involved in pathological processes such as hyperhomocysteinemia where it plays a role in endothelial dysfunction, as well as in cardiac myocytes where it aids in cardioprotection through enhanced preconditioning response to pharmacological agents such as diazoxide [197,198]. However, mitochondrial Cx43 may also contribute to apoptosis of cardiac myocytes, through enhanced release of Ca^2+ ^and cytochrome C from isolated mitochondria, following inactivation of GJIC [199]. Furthermore, connexins have been also localized to the nucleus, and Cx43 has been shown to contain a putative nuclear targeting sequence in its C-terminal domain [187,200,201]. In support of this observation, both full length Cx43 or its C-terminus by itself have been localized to the nucleus, where they inhibit cell growth [202,203].

### Connexin mutations and alterations in disease

Perhaps the significance of connexins in development is best illustrated by the fact that mouse knockouts of Cx26 [204] and Cx45 [205] are embryonic lethal, whereas Cx43 knockouts die at birth due to congenital cardiac abnormalities [206]. Various human diseases have been linked to connexin gene mutations leading to altered gap junction, hemichannel, or general connexin functions [13], and these diseases are divided into seven classes: neuropathic, nonsyndormic and syndromic deafness, skin diseases, cataracts, Oculodentodigital Dysplasia (ODDD), and idiopathic atrial fibrillation [207]. Cx32 mutations are associated with Charcot-Marie-Tooth disease, which affects the central nervous system, leading to reduced myelination and enhanced excitation of neurons [208,209]. Also, mutations in Cx26 may cause either deafness and skin disease, or deafness alone; while the ectopic expression of this connexin in the myoepithelium of the rodent mammary gland disrupts milk ejection (personal communication, Mina Bissell, LBNL, CA). In addition, mutations in Cx30, Cx30.3, and Cx31 have also been implicated in hearing loss and skin disorders [13,210]. Some mutations in Cx26 and Cx32 have been recently mapped to areas of the transmembrane helices of these connexins, suggesting that certain disease-causing mutations may decrease the stability of the connexons [211]. Cataracts can be caused by mutations in Cx46 and Cx50 in the lens fiber cells, leading to lens opacities [212,213]. Cx43 mutations are linked to ODDD [13,214], and these mutations result in non-functional gap junctions and hemichannels in C6 glioma cells [215]. Some ODDD-associated Cx43 mutations result in Cx43 C-terminal truncations, and the skin changes observed in ODDD are correlated with these mutations [216]. Two recent studies have shown that ODDD-associated Cx43 mutations lead to impaired bone differentiation [217] and to delayed development of the mammary gland as well as defective milk ejection in response to oxytocin *in vivo *in a mouse model of the disease [218]. Idiopathic atrial fibrillation is caused by mutations in Cx40, and these result in decreased GJIC either through impaired connexin trafficking or inability to form plaques [219]. It must also be noted that, in addition to connexin mutation-induced diseases, functional connexins and GJIC may play a role in spreading injury, as is seen during hypoxia-ischemia where GJIC spreads neuronal injury [167].

Many connexin-null mouse models of human diseases have recapitulated disease phenotypes and provided crucial insight into these diseases [207]. Such models include the Cx32 knockout which served as a model for Charcot-Marie-Tooth disease, as well as allowed insights into the role of Cx32 in tumorigenesis [220-222]. In addition, in mouse null models of connexins 26, 30, and 31, in which mutations in the human genes are associated with deafness, only Cx26 and Cx30 null mice resulted in deafness [223,224], whereas Cx31 null mice did not [225]. This suggests that connexin-null mouse models may not be the perfect model for studying human diseases characterized by connexin point mutations, and as such, a shift in research towards inserting these point mutations into the orthologous mouse connexin gene has yielded promising results [207,226]. This is further confirmed by connexin-related human diseases, which are lethal, if the relevant connexin is knocked out in rodents, such as Cx43 and ODDD. Such diseases can only be studied by generating mouse models expressing mutated Cx43 [227-229].

Connexins are also implicated in the carcinogenic process, where disruption of normal connexin functions is a hallmark of many tumors [12]. As such, multiple human breast tumor cell lines exhibit down-regulation of connexin gene expression [230], and deficiency of Cx43 gap junctions is considered as a marker of breast tumors [231]. This decreased connexin expression and subsequent decrease in gap junctions is also observed in other tumors, such as gliomas [232]. In contrast to the observed decrease of connexins in the primary tumors, an increase in heterocellular gap junctions with endothelial cells is seen during intravasation and extravasation [233-236]. In addition, Cx43-mediated GJIC results in increased diapedesis of the breast tumor cell line HBL100 [237]. Gap junctional communication has also been correlated with the formation of heterocellular gap junctions between tumor cells and cells of the secondary tumor site or lymph nodes [238-240], whereas other studies state that re-expression of connexins in metastatic tumor cells results in decreased tumor metastatic potential [184,241]. Another study by Saunders *et al*. [242] shows that suppression of MDA-MB-435 tumor cell line metastatic potential results in increased homocellular GJIC, and also in a change in the expressed connexin profile. Untreated cells expressed only Cx32, whereas metastasis-suppressed cells expressed Cx43. This phenomenon is reversed in HuH7 hepatocellular carcinoma cells in which Cx43 enhanced tumor cell malignancy through inhibition of Cx32-mediated GJIC and suppression of Cx32 expression altogether [243], thus, concluding that the impact of GJIC on tumor metastasis may be dependent on the type of connexin expressed and the type of cells used.

## Conclusion

Proper cellular adhesion and interaction, whether between cells or with the ECM, is paramount for proper biological processes. Connexins have important functions in tissue organization, as they, unlike other junctional molecules, mediate the direct transfer of molecules from one cell to another, allowing uniformity and rapid signal transmission. Understanding the biology and regulatory mechanisms of gap junctions at transcriptional, translational, and post-translational levels require further studies to decipher the breadth and complexity of connexin functions. This includes studying the increasing repertoire of Cx-interacting proteins, among which are signaling molecules that are crucial for proper development, such as Wnt signaling *via *β-catenin among others, and for regulating important aspects of connexin function and half-life, as in transport, stability, and degradation.

Furthermore, the recent surge in identifying GJ-independent functions of connexins, whether in the form of hemichannels or in channel-independent roles, has given rise to many novel questions concerning connexin functions in normal physiology. Similarly, this reflects upon the intricacy of connexin alterations and its possible implications in a large variety of diseases. Connexins have been implicated in several disorders and have even been shown to play multiple, if sometimes conflicting, functions in tumor initiation and development. The extent of connexin involvement in cancer, as well as its varied modes of actions must be "reconciled" and ameliorated into a functional map describing their role in different tumor stages. This in turn will yield multiple benefits in the understanding of cell biology in general, and will also provide novel targets and/or mediators of therapeutic strategies for the treatment of different diseases.

## Competing interests

The authors declare that they have no competing interests.

## Authors' contributions

HD and RM drafted the manuscript towards partial fulfillment of their graduate studies. MES and RST supervised the work and finalized the manuscript write up. All authors read and approved the final manuscript.
